# MALDI Mass Spectrometry Imaging for Visualizing *In Situ* Metabolism of Endogenous Metabolites and Dietary Phytochemicals

**DOI:** 10.3390/metabo4020319

**Published:** 2014-05-05

**Authors:** Yoshinori Fujimura, Daisuke Miura

**Affiliations:** Innovation Center for Medical Redox Navigation, Kyushu University, 3-1-1 Maidashi, Higashi-ku, Fukuoka 812-8582, Japan

**Keywords:** mass spectrometry imaging, small molecule, metabolite, phytochemical, food functionality, metabolomics, MALDI, metabolite identification, QSPR

## Abstract

Understanding the spatial distribution of bioactive small molecules is indispensable for elucidating their biological or pharmaceutical roles. Mass spectrometry imaging (MSI) enables determination of the distribution of ionizable molecules present in tissue sections of whole-body or single heterogeneous organ samples by direct ionization and detection. This emerging technique is now widely used for *in situ* label-free molecular imaging of endogenous or exogenous small molecules. MSI allows the simultaneous visualization of many types of molecules including a parent molecule and its metabolites. Thus, MSI has received much attention as a potential tool for pathological analysis, understanding pharmaceutical mechanisms, and biomarker discovery. On the other hand, several issues regarding the technical limitations of MSI are as of yet still unresolved. In this review, we describe the capabilities of the latest matrix-assisted laser desorption/ionization (MALDI)-MSI technology for visualizing *in situ* metabolism of endogenous metabolites or dietary phytochemicals (food factors), and also discuss the technical problems and new challenges, including MALDI matrix selection and metabolite identification, that need to be addressed for effective and widespread application of MSI in the diverse fields of biological, biomedical, and nutraceutical (food functionality) research.

## 1. Introduction

Information on the complex biochemical processes that occur within living organisms requires not only the elucidation of the molecular entities involved in these processes, but also their spatial distribution within the organism. Analytical technologies for elucidating multiple molecular dynamics in the micro-region that retain the spatial information of the target tissue are thought to be indispensable for understanding biological complexity. Chemical stains, immunohistochemical tags and radiolabels are common methods for visualizing and identifying molecular targets. However, there are limits to the sensitivity and specificity of these methods and to the number of target compounds that can be monitored simultaneously. Thus, highly sensitive simultaneous multiple molecular imaging should provide many technical advantages for biological and biomedical researchers.

Metabolites, representative endogenous small molecules, are the result of the interactions of a system’s genome with its environment, and are the end products of gene expression. The metabolome is defined as the total quantitative collection of small-molecular-weight metabolites present in a cell, tissue, or organism, that participate in the metabolic reactions required for growth, maintenance, aging, and normal function [1,2,3,4]. Unlike the transcriptome and proteome that represent the processing of information during the expression of genomic information, the metabolome more closely represents the phenotype of an organism under a given set of conditions and can be defined as the “compound-level phenotype” of the genomic information. Metabolomics, the measurement of the global endogenous metabolite profile from a biological sample under different conditions, can lead us to an enhanced understanding of disease mechanisms, the discovery of diagnostic biomarkers, the elucidation of mechanisms for drug action, and an increased ability to predict individual variation in drug response phenotypes [5,6,7,8]. 

Generally, mass spectrometry (MS) coupled with pre-separation techniques such as liquid chromatography (LC)-MS or gas chromatography (GC)-MS is a conventionally used strategy for investigating metabolomics [9,10,11]. However, these methods are limited in their usefulness for the analysis of tissue samples because of the requirement for metabolite extraction, which results in a loss of information on the spatial localization of the metabolites. In contrast, imaging techniques capable of determining the spatial localization of molecules have revolutionized our approach to diseases by allowing us to directly examine the pathological process, thereby giving us a better understanding of the pathophysiology. In most cases, however, there is a tradeoff among sensitivity, molecular coverage, spatial resolution, and temporal resolution. For example, magnetic resonance imaging (MRI), positron emission tomography (PET), and fluorescence microscopy can visualize the spatial localization of targeted molecules with high sensitivity, but these techniques have low molecular coverage (only a few molecules at a time) [12]. Thus, simultaneous and spatially resolved detection with high sensitivity of a broad range of molecules is still challenging.

**Figure 1:**
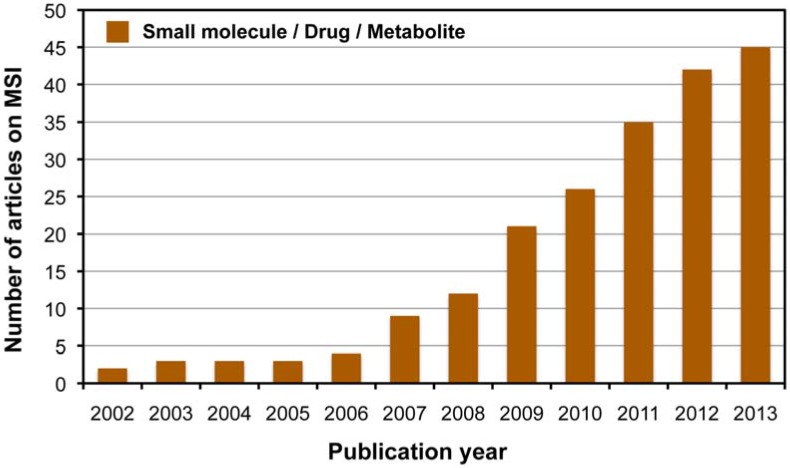
PubMed search results using “mass spectrometry imaging” as the keyword.

MS imaging (MSI) is an effective technology that makes it possible to determine the distribution of biological molecules present in tissue sections by direct ionization and detection. MSI has received considerable attention as a potential imaging technique for a molecular *ex vivo* review of tissue sections from an animal or plant based on label-free tracking of endogenous or exogenous molecules with spatial resolution and molecular specificity [13,14,15]. The matrix-assisted laser desorption/ionization (MALDI)-MSI technique was initially developed as a tool for intact protein imaging from the tissue surface [15,16,17,18,19]. In current research, proteins or peptides are still the primary targets of this imaging technique [20]. However, MSI analysis of a wide variety of low-molecular-weight compounds including endogenous metabolites and drugs has gradually increased (Figure 1). In this review, we describe recent advances and difficulties in developing an analytical platform for MSI of endogenous metabolites or dietary phytochemicals (food factors).

## 2. MALDI-MSI for Visualization of Endogenous Metabolite Distribution

**Figure 2:**
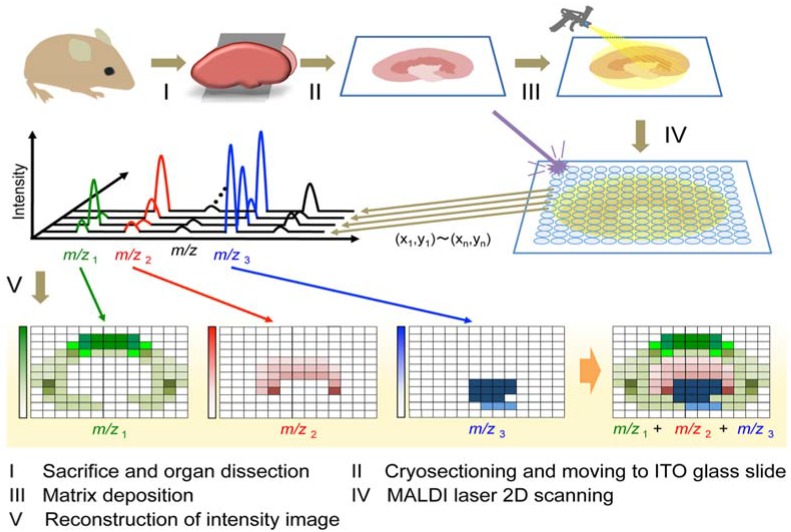
The schematic representation of MALDI-MSI experimental procedures.

MALDI, a commonly available ionization method used for MSI, is a laser desorption ionization (LDI) method that softly ionizes several biological molecules. The workflow of MALDI-MSI is shown in Figure 2. It is comprised of tissue preparation, matrix application, MSI data acquisition, followed by data analysis and image construction. This ionization technique is usually combined with time-of-flight (TOF)-MS. A conventional MALDI source is equipped with a UV laser such as a nitrogen laser (337 nm) or Nd-YAG (355 nm). MALDI-MSI is typically performed at spatial resolutions of 10–200 μm in single organs. The spatial resolution is primarily dependent on the diameter of the laser irradiated area which is usually more than 5 µm [21]. However, because MALDI-MSI requires a matrix application step, diffusion of metabolites within the tissue during matrix application and the heterogeneous size of crystal formation may also limit the spatial resolution. Generally, matrix application is performed by spray coating [22,23,24] or droplet printing deposition [25,26]. Spray deposition is typically faster and offers higher spatial resolution, but the amount of solvent must be carefully controlled to prevent the tissue becoming overly wet. The droplet deposition method sacrifices resolution, which is typically no better than 200 μm because of the size of the matrix droplets. However, in this droplet deposition method, sensitivity is high because of the high analyte extraction efficiency of the droplets and there is no risk of analyte delocalization outside of the matrix spot. When applying the matrix dissolved in solvent, it is critical that the matrix spray is wet enough to extract the analytes from the tissue and into the surface matrix crystals, but not so wet that the analytes will delocalize from their original positions to neighboring regions, leading to a loss of image spatial integrity. In contrast, dry matrix application methods have been reported for imaging small molecules in tissues, which minimize potential delocalization [27,28]. Vapor-phase deposition of the matrix through sublimation produced a homogeneous coating of matrix across the tissue section [29,30,31]. These experiments showed a significantly enhanced signal for lipids, reduction in laser spot-to-spot variation of secondary ion yield, as well as reduction in alkali metal contamination [32]. Sublimation has the desired effect of purifying the matrix of any nonvolatile impurities during the coating process [29]. On the other hand, this method showed only poor sensitivity, due to a lack of incorporation of the analyte into the matrix [29]. To overcome this issue, Spengler *et al.* separated the matrix preparation procedure into two independent steps, leading to an improved sensitivity and spatial resolution [33]. The first step is a dry vapor deposition of matrix onto the sample. In a second step, incorporation of analyte into the matrix crystal was enhanced by controlled recrystallization of matrix in a saturated water atmosphere. This approach achieved an effective analytical resolution of 2 μm for scanning microprobe MALDI-MS. Recent work has also demonstrated the utility of ionic liquid matrices for MALDI-MSI [34,35]. These matrices are advantageous in that there are no crystals to limit the spatial resolution. 

During its first decade of use, MALDI-MS was employed for synthetic polymer or protein (peptide) analysis. In the post-genomic era, the dramatic progress of bioinformatics research accelerated the use of MALDI-MS in proteomics research for identifying vast numbers of proteins [36]. MALDI-MS is a highly sensitive analytical method that can be used to analyze low concentrations (~ fmol) of tryptic peptides. Sensitivity is an extremely important parameter for MSI because numerous biological molecules exist in very small quantities on a thin tissue section. However, MALDI-MS has rarely been used for low-molecular-weight metabolite analysis because many kinds of matrix and/or matrix-analyte cluster ion peaks are observed in the low-mass range (*m/z* < 700), and the strong peaks that they generate interfere with the detection of the target low-molecular-weight compounds.

Based on these early observations, lipid molecules became the first targets for MSI studies of endogenous metabolites because the *m/z* range of most lipid molecules was more than 700. Lipids are also abundant in tissues (*e.g.*, more than 60% dry weight of brain tissue) and are easily ionized because of the presence of a polar head [21,37,38,39]. For example, glycerophospholipids such as phosphatidylcholine, phosphatidylethanolamine and phosphatidylserine (PS), which have positively charged polar heads, were detected in the positive ion mode. On the other hand, glycerophospholipids such as phosphatidylinositol (PI) and phosphatidylglycerol, which have negatively charged polar heads, were detected in the negative ion mode. In addition, glycosphingolipids such as gangliosides, sulfatides (ST), and galactosyl-ceramide were visualized by MALDI-MSI. These lipids can easily ionize, and their region-specific distribution on tissue sections has been observed using traditional matrices such as 2,5-dihydroxy benzoic acid (DHB), α-cyano-4-hydroxycinnamic acid (CHCA), and sinapinic acid (SA). In response to extracellular stimuli, several of the fatty acids in the phospholipids are released and converted into bioactive lipids that mediate important biological processes [40]. Thus, information on the unique distributions of the various phospholipids has contributed to a better understanding of the molecular basis of diverse biological phenomena [41].

Alternatively, a smaller number of researchers have tried to apply MALDI-MSI to investigate smaller endogenous metabolites. Metabolites in the low-mass range (*m/z* < 700) with distinct distributions in various tissues were searched from a massive forest of background peaks that were generated as a result of using conventional matrices such as DHB and CHCA. The representative low-molecular-weight metabolites from animal and edible plant tissues were shown in Table 1. Heme B (*m/z* 616) [42], GABA (*m/z* 104) [43], and α-tocopherol (*m/z* 431) [44] have been successfully detected, and their unique distributions on the surface of the plant and animal tissue sections have been visualized. However, the low ionization efficiency and interference of matrix peaks from the use of conventional matrices have made it difficult to detect other metabolites. Recently, 9-aminoacridine (9-AA) was reported as a suitable matrix for low-molecular-weight metabolite analysis [45]. When 9-AA was used in negative ion mode, only a few peaks derived from the matrix were observed in the low-mass range (*m/z* ~500). In addition, the excellent ionization efficiency of 9-AA for important cellular metabolites (in the order of attomoles) was demonstrated [46,47]. Using the 9-AA matrix, several endogenous metabolites were detected from extracts of *Escherichia coli* and yeast [48,49,50]. Shroff *et al.* succeeded in visualizing the distribution of antiherbivore glucosinolates in *Arabidopsis thaliana* leaves using 9-AA [51]. The results indicated that there were differences in the proportions of the three major glucosinolates in different leaf regions, and that their distributions appeared to control the feeding preference of the *Helicoverpa armigera* larvae. Benabdellah *et al.* reported that the location of 13 metabolites in the normal rat brain, almost all of which were nucleotide derivatives, could be observed using MSI [23]. We recently showed, for the first time, the applicability of MALDI-MS for obtaining chemically diverse metabolite profiles on a single-mammalian cell [52]. Human HeLa cells mounted on indium tin oxide (ITO) glass were imaged using 9-AA. Negative ion mode MALDI-MS spectra corresponding to 50 individual signals were collected, and ATP, fructose-1,6-bisphosphate, and citrate were successfully identified as the representative metabolites [52]. This result indicated that the 9-AA-MALDI-MSI system allowed single-cell metabolomic analysis to be successfully performed. Furthermore, the ultra-sensitive MALDI-MS technique enabled the spatially resolved detection of a broad range of metabolites with unique distributions, and helped in the identification of more than 30 metabolites that included nucleotides, cofactors, phosphorylated sugars, amino acids, lipids, and carboxylic acids in normal mouse brain tissue. The application of this technique and metabolic pathway analysis to a rat transient middle cerebral artery occlusion (MCAO) model allowed visualization of a spatiotemporal behavior of metabolites in the central metabolic pathway regulated by ischemia-reperfusion [52]. 

**Table 1:** Published literature describing the application of MALDI-MSI for endogenous metabolites and dietary phytochemicals in animal and edible plant tissues.

Matrix	Analyte	Tissue	Species	Ref.
**Animals**				
DHB	Heme B	Liver	Human	[42]
	Acetylcholine	Brain	Mouse	[53]
9-AA	Nucleotides, sugar phosphates	Brain	Rat	[23]
	Nucleotides, sugar phosphates, organic acids, amino acids	Brain	Rat, Mouse	[52]
1,5-DAN	EGCG and its phase II metabolites	Liver, Kidney	Mouse	[54]
**Edible plants**				
DHB	γ-Oryzanol, α-tocopherol, phytic acid	Grain	Rice	[44]
	GABA, amino acids, sugars	Fruit	Eggplant	[43]
	Glycoalkaloids	Tuber	Potato	[55]
	Anthocyanins	Fruit	Blueberry	[56]
CHCA	Oligosaccharides	Stem	Wheat	[57]
	Amino acids, sugars, sugar phosphates	Grain	Wheat	[58]
	Flavonoids, dihydrochalcones	Fruit	Apple	[59]
9-AA	Amino acids, sugars, sugar phosphates	Grain	Wheat	[58]

**Figure 3:**
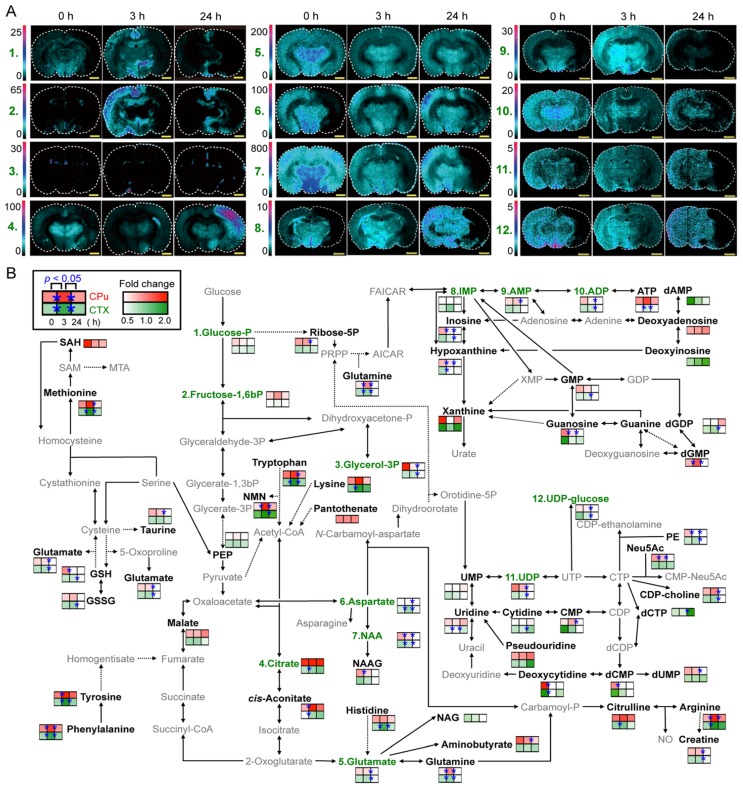
Integrated MSI and LC-MS techniques for visualizing spatiotemporal metabolite distribution. (**A**) *In situ* MSI visualized the spatiotemporal metabolic behavior in MCAO rat brain. Metabolites (nucleotide and amino acid metabolism, as well as the central pathway) were simultaneously visualized in a single MSI experiment. *Scale bar* (1.0 mm). (**B**) Comparative visualization of the central metabolic pathway and its peripheral pathways in the CPu (*upper box, red*) and CTX (*lower box, green*), as determined by LC-MS. Significant differences (Student’s *t*-test, *p<0.05) are indicated by *asterisks* on the *colored boxes*. *Black* and *grey letters* indicate LC-MS-detected metabolites and unmeasured metabolites, respectively. *Green letters* indicate metabolites detected by both LC-MS and MSI. *Solid arrows* represent a single step connecting two metabolites, and *dotted arrows* represent multiple steps. Adapted with permission from [60]

Generally, it is known that the molecular coverage of MSI is lower than that of LC-MS and GC-MS [60,61]. An additional understanding of the dynamics of more comprehensive metabolites detected by other MS platforms with higher coverage, which cannot be detected by MSI, may lead to further elucidation of the complex pathological mechanisms underlying various diseases. Irie *et al.* showed that an integrated strategy combining MSI (spatial information but low coverage) and its complementary technique LC-MS (high coverage but loss of spatial information) was effective for visualizing diverse spatiotemporal metabolic dynamics, a MCA-regulated metabolic change in several metabolic pathways including pyrimidine-, amino acid- and TCA cycle-related metabolism, during pathological progression (Figure 3) [60]. Hattori *et al.* have also reported spatiotemporal changes in energy charge, adenylates, and NADH during focal ischemia in a mouse MCAO model by combination of MALDI-MSI and capillary electrophoresis (CE)-MS [61]. These findings highlight the potential applications of the *in situ* metabolomic imaging technique to visualize spatiotemporal dynamics of the tissue metabolome, which will facilitate biological discovery in both preclinical and clinical settings.

The aforementioned MSI techniques involve sacrificing animals that are not suitable for metabolic investigations of brain *in vivo* because the organ is susceptible to postmortem changes in concentrations of labile energy metabolites [62]. Particularly, molecules with high-energy phosphates including glucose intermediates and phospho-nucleotides, are highly sensitive to the postmortem degradation [61]. To overcome this issue, Sugiura *et al.* used a head-focused microwave irradiation method, which can stop brain metabolism within 1 s [62]. This new method allowed us to quantify and to visualize brain metabolic flux of glucose into specific metabolites *in vivo*. High quality MSI requires proper preparation of tissue specimens to provide reproducibility and high ionization efficiency. There are many recent research articles and reviews discussing sample preparation procedures [62,63,64,65].

Taken together, these findings show great potential for the realization of “metabolomic imaging” using MALDI-MSI. Although the present MALDI method is highly sensitive and well established on the MSI platform, some limitations remain to be overcome before broad endogenous metabolite imaging can be achieved [66]. In MALDI, the detection of molecules is completely dependent on the matrix. In addition, the crystal size of the deposited matrix strongly affects both experimental reproducibility and spatial resolution in MALDI-MSI. To accelerate the use of a MALDI-based metabolite imaging platform, substantial progress in matrix development and its application is required. In MALDI-MSI, because damage of the biomedical tissue section induced by laser irradiation is relatively modest, histological and biochemical evaluations can be performed on the same tissue slice after the MSI experiment is complete. The additional information that can be obtained using this approach allows highly precise and reliable molecular pathological evaluation of the results based on combining different imaging modalities, MSI and other forms of pathological and biochemical imaging. 

## 3. Visualization of Dietary Phytochemical to Understand Its *In Situ* Metabolism

MSI is a rapidly emerging technology for visualizing the localization of exogenous drugs and their metabolites in biological tissues [67]. It has several attractive advantages over traditional imaging and analytical methods used in pharmaceutical research, especially the fact that the technology is completely label free. However, careful consideration is essential for the choice of methodology because drugs and metabolites are often more difficult to analyze in biological tissues than endogenous species because of their relatively low abundance. 

An important early phase of drug discovery is determining how a candidate drug is distributed and metabolized within the body. Conventionally, spatial information on compound distribution in whole animals is obtained by whole-body autoradiography (WBA) (typical spatial resolution 100 μm) and microautoradiography (MARG) for more detailed imaging of smaller tissues (typical spatial resolution 10 μm) [68]. In these methods, the pharmaceutical compound is labeled with radiative nuclide replacing the nonradioactive one prior to dosing, and this radiolabel is visualized in the tissue section of the animal. This advantage is the highly sensitive and fully quantitative nature of the analysis, but there are several disadvantages. The parent drug cannot be distinguished from its metabolites even if the metabolite contains the radiolabel. The synthesis of the drug compound with the incorporated label is an expensive and frequently time-consuming process. Furthermore, it can take several days to several weeks of exposure time to develop radiographic images of sufficient sensitivity for distribution studies. In contrast, PET is an alternative *in vivo* imaging technique for dosed pharmaceuticals. In this approach, the drug is radiolabeled prior to administration. The advantages of PET are true *in vivo* imaging and the ability to follow the drug distribution in real time. However, specificity is again an issue as any metabolites cannot be distinguished from the parent drug. In addition, PET suffers from relatively poor spatial resolution (approximately 1 mm for small animal studies) but has the significant advantage of being fully quantitative [69]. MSI can provide information on the specific localization of the analyte of interest comparable to WBA. Thus, MSI allows for detailed localization of the parent compound and its metabolites to be determined in a single experiment without any labeling. It also offers the unique ability to co-localize drug distribution signals with endogenous analytes of interest as biological markers of disease progression, therapeutic effect or toxicology.

Natural products derived from medicinal plants are an abundant source of biologically active phytochemicals, many of which have formed the basis for development of pharmaceuticals and nutraceuticals [70]. To date, imaging of small molecules including different classes of primary and secondary metabolites is the most frequent applications of plant-targeted MSI [58,71,72]. These studies will encourage an increased understanding of diverse plant biological systems and increase applications in breeding, crop improvement, and functional food design [44,56,71,73,74]. Differential distribution patterns have been evaluated for a number of molecular species, namely, lipids, amino acids, and sugars, as well as highly abundant secondary metabolites, such as polyphenols, anthocyanins, alkaloids, and glucosinolates from a variety of plant species [73]. The representative molecules from edible plants were listed in Table 1. However, there is little information on the use of MSI to follow *in vivo* administration of these and other bioactive dietary phytochemicals to animals. In most cases, the aforementioned dietary compounds and drugs have been detected by MALDI-MS using traditional matrices such as DHB, CHCA, SA, and 9-AA [67,73]. These matrices are certainly effective for MALDI-MSI of limited drugs in tissue sections, but such matrices cannot be used to easily visualize the localization of many dosed dietary compounds (food factors), including phytochemicals and their metabolites, due to their low abundance in the target tissue as well as interference with background peaks from the matrix and endogenous molecules. For effective ionization of the analyte in MALDI-MS, the optimum matrix needs to be determined because there is often no direct correlation between the choice of matrix and its ability to ionize a bioactive small molecule of interest. Kim *et al.* screened 41 chemicals as potential matrices for the representative bioactive dietary phytochemical, epigallocatechin-3-*O*-gallate (EGCG) [54]. EGCG is the most abundant polyphenol in green tea (*Camellia sinensis* L.). Many studies have revealed that the representative food factor, EGCG, possesses various pharmacological properties, such as anti-cancer, anti-atherosclerosis, anti-obesity, and neuroprotective effects [75,76,77,78,79,80]. To elucidate the precise mechanism underlying the bioactivity of this dietary polyphenol, spatiotemporal information is needed. Although some studies have visualized its tissue distribution by fluorescence imaging, cerium chloride staining, and radioactive labeling assays [81,82,83], spatiotemporal information has been lacking because of the absence of an analytical technique that can easily detect the localization of the naïve polyphenol. Conventional molecular imaging generally requires labeling steps that are time-consuming, expensive, and labor-intensive. In addition, the ability of these techniques to allow the discrimination of molecules is insufficient for simultaneous visualization of a target compound and its metabolites. It is expected that the use of MSI can overcome these issues, but the development of such a technique has been a challenge. For example, MALDI-MSI screening of EGCG showed that the EGCG peaks were not observed with DHB, CHCA, SA, or 9-AA [47], which are the most effective major matrices for ionizing small molecules [42,43]. However, 1,5-diaminonaphthalene (1,5-DAN), harmane, norharmane, harmine, and ferulic acid all allowed for the detection of EGCG (*m/z* 457 [M–H]^−^) in negative ion mode without any background peak interference [47]. Furthermore, among such candidate chemicals, only 1,5-DAN was useful to visualize the distribution of a single oral dose of EGCG (2,000 mg/kg b.w.) in mouse tissue sections. Provided that chemical screening data are available online, this information may be useful for matrix selection and development for highly sensitive detection of EGCG or its derivatives, and structure-based matrix screening for MSI of dietary polyphenolic compounds. 

**Figure 4:**
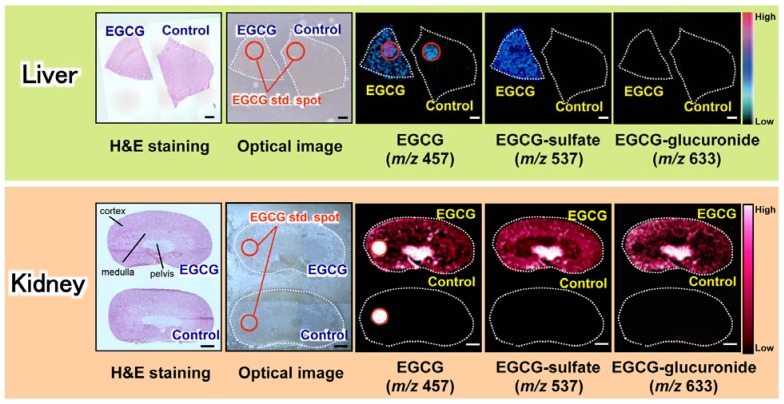
Visualization of dosed EGCG and its phase II metabolites in tissue micro-regions. Simultaneous visualization of EGCG and its phase II metabolites in liver (upper panel) and kidney (lower panel) sections. Three different images are shown in hematoxylin and eosin (H and E) staining, optical microscopy, and MALDI-TOF-MS: EGCG (*m/z* 457), EGCG-monosulfate (*m/z* 537), and EGCG-monoglucuronide (*m/z* 633). An additional EGCG spot (red circle) was visualized as the positive and internal control. Adapted with permission from [54].

Understanding the metabolic fates of bioactive dietary polyphenols is indispensable for determining their *in vivo* molecular mechanisms [76]. Some studies have reported that green tea polyphenols are subjected to phase II biotransformation and predominantly undergo methylation, glucuronidation, and sulfation in the intestine, liver, and kidneys [84]. However, both the functions of the metabolites and their localizations in different tissue micro-regions were unclear [85]. In contrast, 1,5-DAN-MALDI-MSI was able to visualize a spatially resolved biotransformation based on simultaneous mapping of orally dosed EGCG and its phase II metabolites such as its monosulfate (*m/z* 537) and monoglucuronide (*m/z* 633) forms (Figure 4) [54]. Interestingly, unlike liver, the localization patterns in the kidney compartments (pelvis, medulla, and cortex) were clearly different among EGCG and its phase II metabolites. In the kidney tissue extract, EGCG and its major conjugates (methylated, sulfated, and glucuronidated forms) were observed. The peak abundance of such three conjugates was markedly lower than that of EGCG. Nevertheless, both sulfated and glucuronidated forms were detected in MALDI-MSI measurements, but there was no peak of methylated form (*m/z* 471 [M–H]^−^). In negative ion mode MALDI-MS, the phosphorylated compounds and carboxylic acids were efficiently ionized, indicating that compounds with leaving groups, including phosphate and carboxylic groups, readily undergo deprotonation [47,48,52,86]. Unlike methylation, sulfation or glucuronidation can introduce a leaving group (sulfate or carboxylic group, respectively) into EGCG. Therefore, in negative ion mode MALDI-MS using 1,5-DAN, the introduction of such an ionizable group may contribute to preferable ionization, higher MALDI efficiency, of EGCG phase II conjugates in spite of their lowered tissue abundance compared to EGCG [54]. Although the bioavailability of EGCG is very low [76,84], this sensing technology was first able to visualize the *in situ* distribution of EGCG phase II metabolites in liver and kidney sections after oral dosing. The use of 1,5-DAN-MALDI-MSI will open new avenues for investigating the *in situ* metabolism of a bioactive dietary polyphenol subjected to phase II biotransformation, and may help to accelerate the highly effective and efficient design of plant-derived pharmaceuticals, multicomponent botanical drugs, dietary supplements, and functional foods. The advantages of this MALDI-MS methodology include label-free imaging and simultaneous detection of an orally dosed dietary polyphenol and its metabolites. 

However this technique could not be used to visualize EGCG and its phase II metabolites in both kidney and liver tissue sections after oral dosing at a normal intake level (20 mg/kg b.w.) [54]. For overcoming the limitation of the 1,5-DAN-MALDI-MSI technique and for its practical use, further improvement of the detection sensitivity, such as the targeted selective reaction monitoring mode (SRM) approach using QqQ [87], the application of a Fourier transform ion cyclotron resonance-MS (FT-ICR-MS) instrument capable of accumulating continuously selected ions [88], and on-tissue chemical derivatization approaches to increase the ionization efficiency [89,90] as well as the improvement of MALDI efficiency based on matrix choice and development, may be required. In addition, other studies regarding kinetic histochemistry [67,91,92], microscopic analysis with high-spatial resolution [21,93,94], 3D imaging [95,96,97], and distribution of other metabolites [84,97] will be required to unravel both the biological consequences of biotransformation of the dietary polyphenol and its mechanism(s) of action.

## 4. Metabolite Identification Strategy Using Ultrahigh-Resolution MS

When MALDI-MSI experiments are performed, tens or hundreds of peaks are simultaneously detected in a single run. Understanding their unique distributions in a tissue section should provide important information. However, the crude analytical data without peak identification is difficult to interpret, and discovery of biomarkers or elucidation of biological and disease mechanisms is hindered. Conclusive identification of a target compound and its metabolites in tissues often becomes problematic because of the presence of many interfering peaks from endogenous species and matrix within the low mass range. The comprehensive or targeted measurement of endogenous metabolites or an exogenous small compound and its metabolites extracted from whole tissues is usually carried out by conventional analytical methods such as LC-MS or GC-MS. Each molecule is normally identified by comparing two-dimensional data of the observed peak, its retention time and either the exact *m/z* or its MS/MS spectrum, with that of an authentic standard peak. In contrast, MSI experiments can be used to directly analyze a series of small molecules on the surface of a tissue without employing the extraction and separation processes that are required for the use of conventional methods. Thus, the identification of small molecules must be performed by analysis of only one-dimensional data, the exact *m/z* or the MS/MS spectrum of the observed peak. 

Generally, an MS/MS approach is applied to identify metabolites in MSI experiments [23,98]. In-house library or public database (for example, Mass Bank [99], the METLIN database [100] and/or the Human Metabolome Database [101]) search strategies using the available MS and MS/MS mass spectra patterns of known and available compounds are usually used as the well-established chemical annotation of the experimental MS data. This strategy is not data-driven; rather, it is completely dependent on the spectral databases and comparisons with reference authentic standard spectra. There are several problems associated with using these methods. First, the commercially available compounds do not include all biological metabolites because there are thought to exist large numbers of unknown metabolites [66]. Second, poor mass resolution can result in the peaks of several metabolites overlapping; in such cases, the MS/MS spectrum becomes a mixed spectrum of overlapping metabolites. TOF-MS (resolution ~ 50,000) is widely used for MSI experiments; however, their mass resolution power is low. These limitations make it difficult to identify individual peaks in MSI spectra using an MS/MS strategy. Indeed, only 20%–30% of all peaks detected from mouse brain tissue were successfully identified by an MS/MS approach [52]. Clearly, compared with the ability of an MS/MS approach to identify proteins, which is usually performed by Mascot algorithm [102], the MS/MS approach alone is not effective enough to identify metabolites in target tissues. In addition, there is the basic problem regarding the low power of MS in selecting precursor ions. Typically only 1 Da window is used, in which several precursors are selected and providing chimera MS/MS spectra, similar to proteomics [103,104]. The identification of various biological metabolites on the basis of MS data still remains a challenging issue.

**Figure 5:**
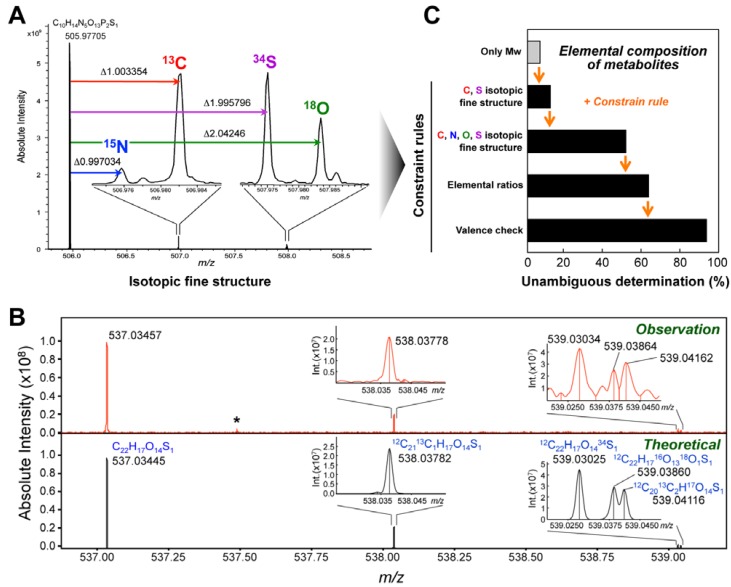
Isotopic fine structure analysis for unambiguous determination of metabolite ECs. (**A**) Mass spectral observation of PAPS (C_10_H_15_O_13_N_5_P_2_S_1_) by high-resolution ESI-FT-ICR-MS in negative ion mode. Multiplet isotopic peaks were observed in the (M–H^+^+1)^−^ and (M–H^+^+2)^−^ regions, and these peaks were successfully assigned to the substitution of a stable isotope for each element. (**B**) Isotopic fine structures were measured by MALDI-FT-ICR-MS in a liver tissue section after oral dosing of EGCG. Theoretical peaks of EGCG-monosulfate (C_22_H_17_O_14_S_1_) are shown in negative ion mode, and isotopic peaks were also observed. Asterisk shows background peaks. (**C**) Improvement of the accuracy for determining one correct EC by applying the additional constraint rules. The rate of the identification of 1 candidate EC obtained for metabolites estimated by using only the MW, isotopic peaks of C and S elements, isotopic peaks of C, N, O and S elements, element ratios (based on the Seven Golden Rules and the O/P ratio) and the LOUIS and SENIOR check (introduced in the Seven Golden Rules). Partially adapted with permission from [54,115].

The elemental composition (EC) of an unknown metabolite is one of the most important pieces of information for reliable structure determination [105,106,107,108]. Several researches have focused on the direct determination of EC directly from mass spectra [109,110,111]. In principle, one EC has one molecular weight and one molecular weight determines one EC; however, this relationship strongly depends on the accuracy of mass measurement. In fact, many hundreds of candidates based on the EC of metabolites are found at lower accuracy. Mass spectra with high accuracy (<1 ppm) effectively narrow down the number of candidates based on the EC [112]. Furthermore, the EC can be effectively determined by combining an accurate *m/z* value and MS^n^ spectra [110]. Recently, it was reported that ultrahigh-resolution (resolution > 500,000) MS analysis, using FT-ICR-MS, enabled the EC to be directly determined using isotopic fine structure (Figure 5A) [113]. High-resolution MS provides another advantage for MSI because low mass resolution causes the critical problem that an observed imaging map of a single *m/z* value is at high risk of containing overlapping images of proximate multiple metabolite peaks.

In low mass resolution MSI, individual metabolites can be detected by MS/MS fragment imaging [114]; however, because this is a completely targeted analysis, a preliminary MS/MS experiment of a standard of the target molecule is required beforehand. Very close mass peaks that are observed as a single overlapped peak by low mass resolution MS can be separated clearly and visualized as independent multiple images by ultrahigh-resolution MSI [14]. However, digital data from a single ultrahigh-resolution MSI experiment will be over a terabyte in size. Furthermore, the primary issues are the length of time taken to acquire the MS images *versus* QqQ and QqTOF in addition to very expensive (>1 million EUR). For these reasons, MALDI-FT-ICR-MSI is not routinely applied to high-throughput tissue imaging and most applications to date have focused on small tissue areas or individual organs [67]. Also, the ICR cell has a limited ion capacity, which prevents sensitive measurement of low-level compounds in the presence of highly abundant endogenous metabolites. This can be avoided to some degree by selecting narrow mass ranges for analysis. Researchers must choose the MS platform for MSI that best matches the experimental circumstances and purpose.

In many cases, *in vivo* administered drugs and food factors can prove more difficult to analyze in the target tissues than endogenous small molecules because of their relatively low abundance. In addition, the concentration of the dosed compound in a tissue section may not be measurable because of the strong dependence of the MS signal in MALDI-MSI on the chemical and biological environment [67,116]. Unlike an isolated drug in a solution deposited on a steel target, a molecule trapped in a tissue undergoes many interactions. Molecules also experience an ionization competition phenomenon linked to the other compounds present in its immediate environment. The combination of these parameters must be taken into account when interpreting MSI. In fact, certain drugs (propranolol, olanzapine, and imipramine) [116,117] and the dietary phytochemical EGCG [54] underwent ion suppression in some tissues (brain, lung, kidney, or liver), and their limits of detection were remarkably lowered. This ion suppression effect also makes it difficult to identify the peak of interest in tissue sections by MS/MS because of the diminished peak signal. In addition, if the reference standard is not commercially available, the peak identification by low-resolution MS such as TOF-MS cannot be performed. In contrast, ultrahigh-resolution MALDI-FT-ICR-MS can be used to unambiguously determine the EC of low abundance compounds in tissue sections on the basis of the relative isotopic abundance (RIA) of ^13^C, ^18^O, and ^34^S, without the requirement of MS/MS in a standard-independent manner (Figure 5B) [54].

In MS-based metabolomics studies, reference-free identification of metabolites is still a challenging issue. It was reported that the EC of metabolites could be unambiguously determined using an isotopic fine structure observed by ultrahigh-resolution FT-ICR-MS, which provided the RIA of ^13^C, ^15^N, ^18^O, and ^34^S [113]. Most recently, the efficacy of RIA for determining the ECs based on the MS peaks of metabolites was evaluated by using the mass spectra of 20,258 known metabolites that were simulated with ≤ 25% error in the isotopic peak area to investigate the error size effect of isotopic peaks on the rate of unambiguous determination of ECs [115]. The simulation indicated that in combination with the reported constraint rules, RIA led to unambiguous determinations of the ECs for more than 90% of the tested metabolites (Figure 5C). It was noteworthy that the processing could distinguish alkali metal-adducted ions ([M + Na]^+^ and [M + K]^+^) in positive ion mode. However, a significant and remarkable decrease of the EC determination performance was observed when the method was applied to experimental metabolomic data (mouse liver extracts analyzed by infusion ESI), due to the influence of noises and biases on the RIA. To achieve the ideal performance indicated in the simulation, an additional method was developed to compensate biases in the measurement of ion intensity [115]. The method improved the performance of the calculation to determine ECs for 72% of the observed peaks. The proposed method should be a useful starting point for high-throughput identification of metabolites in metabolomic research. During the experimental evaluation of the method, it was found that the dynamic range of the quantitative performance of the MS analysis was the limiting factor for the performance of the calculation, a factor which was partially relieved by numerical post-processing. Assuming that the ion motion in the ICR cell was relevant, improvement in the design of the ICR cell is expected to overcome the issue. A preliminary sample separation, *e.g*. the use of LC, is also a way to reduce the amount of ions introduced into the cell at any one time point. Since the critical requirement of the EC calculation is ultra-high mass resolution (R > 300,000), other types of instrumentation with ultra-high mass resolving power, *e.g*. Orbitrap MS, may be suitable for the RIA-based calculation of ECs. 

## 5. The Rational Understanding of MALDI Ionization

It is well known that the scope of detectable compounds from MALDI-MS analysis is strongly associated with the molecular species of the matrix. To date, extensive research has contributed to elucidating the fundamental mechanism of MALDI [118]. However, to clarify whether a target molecular species can be sensitively detected by MALDI-MS, an experimental trial is still required because there is currently no decisive rationale to predict which compounds will be ionizable with which matrices. This problem is largely attributable to the chemical and structural diversity of metabolites which hinders the rational understanding of the interrelationships between the metabolites and the potential factors affecting their ionization. Therefore, modeling the potential relationship between the structural properties of the metabolites and their ionizability during MALDI should prove useful.

In targeted analyses, the merit of property modeling lies in the prediction of the probability of the ionization of metabolites yet to be analyzed in MALDI-MS. In non-targeted analysis, the model would work to screen chemical structures plausibly assigned to a detected peak, even if compounds with similar *m/z* values are not distinguishable. Furthermore, the expected signal response calculated from the ionization efficiency model would provide insights into the abundance of the compound of interest. As a practical case study, Yukihira *et al* attempted to extract structural properties of metabolites that contribute to their ionization in MALDI-MS analyses using 9-AA as the matrix [86], because it is one of the most frequently used matrices for metabolite analyses by MALDI-MS [66], and has been proven to be applicable for MALDI of a series of metabolites in the central metabolic pathway, such as phosphorylated compounds and carboxylic acids. The 9-AA-MALDI-MS have been utilized for various studies, including high-throughput and highly sensitive metabolite analyses [37,46,47,48] as well as metabolite MSI [23,30,52,60,66,119,120].

**Figure 6:**
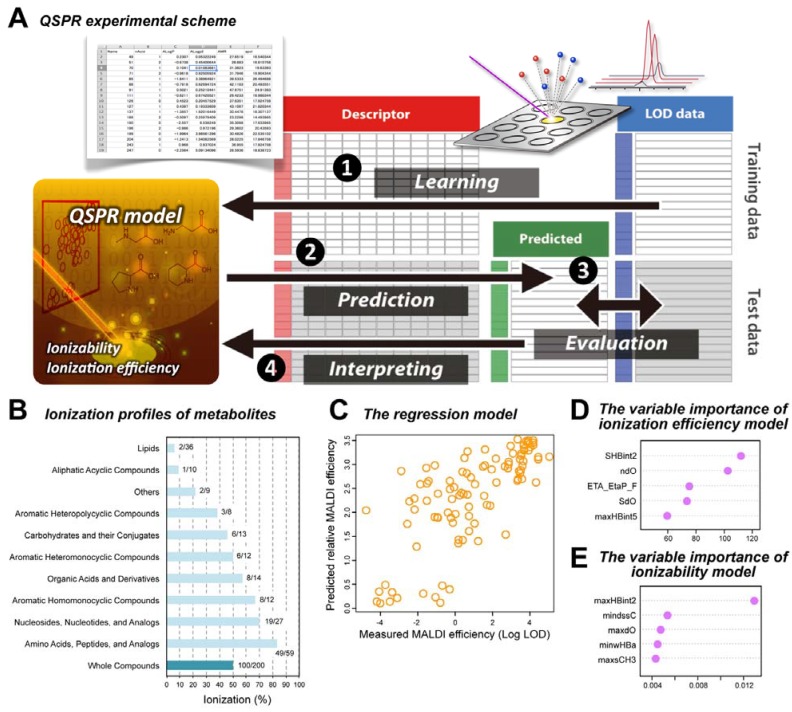
A strategy for a QSPR approach for MALDI ionizability and ionization efficiency of metabolites. (**A**) QSPR experimental scheme (**B**) Ionization profiles of 200 standard metabolites. (**C**) The Random Forest regression model for the ionization efficiency in 9-AA-MALDI. The variable importance, molecular descriptors contributed to model construction, of (**D**) ionization efficiency and (**E**) ionizability models. Partially adapted with permission from [86].

To cover a wide range of structural diversity and biological importance, 200 metabolite standard compounds were selected, and their ionization profiles (both the ionizability and ionization efficiency) in 9-AA-MALDI-MS were examined [86] (Figure 6). Interestingly, a distinct ionization profile was observed even for compounds with a similar structure (*e.g.*, alanine and β-alanine, or leucine and isoleucine). In these cases, β-alanine and isoleucine exhibited concentration-dependent peak intensity in MALDI-MS analysis, whereas alanine and leucine were not detected. Generally, structural similarity of low-molecular-weight compounds should give similar physicochemical properties. However, the observations strongly indicated that the apparent properties of the molecule, such as the presence of functional groups, are insufficient to explain the diverse ionization profiles of the compounds. The physicochemical factors of the metabolites that influenced the ionization profiles were also of interest. To address these factors, Yukihira *et al.* performed non-hypothesis-based statistical modeling, where the source of efficient MALDI was sought by molecular descriptors of target compounds [86]. Using the ionization profile dataset, a quantitative structure–property relationship (QSPR) analysis was performed to model the experimental evaluation using *in silico* molecular descriptors of the metabolites, calculated by the PaDEL-Descriptor software program [121]. As there were hundreds of descriptors available, the Random Forest method was employed because of its robust applicability to large multivariate datasets and unbiased modeling performance [122]. The classification model for the ionizability achieved a 91% accuracy, and the regression model for the ionization efficiency reached a rank correlation coefficient of 0.77. An analysis of the descriptors contributing to this model construction suggested that proton affinity is a major determinant of the ionization, whereas some substructures hinder efficient ionization. 

In contrast to empirical approaches, the aforementioned rational and predictive strategy employed a systematic analysis of the ionization profiles from 9-AA-MALDI-MS for the first time [86]. For MALDI-MS analysis, the ionizability prediction model allows evaluation of the likelihood of peak identification while the ionization efficiency model should help to estimate the abundance of the metabolite based on the observed signal intensity. The relevant descriptors can be interpreted as the structural preference specific to 9-AA and/or negative ion mode MALDI-MS analysis. The QSPR approach should also be applicable for other MALDI matrices to characterize the structural properties of target compounds for preferred ionization. Recently, Shroff *et al.* reported a rational protocol for MALDI matrix selection based on Brønsted–Lowry acid–base theory and density functional theory (DFT) quantum chemical calculations to study a wide variety of metabolites [123]. Several important physicochemical characteristics for the rational design of matrices are proposed: negative ion-mode matrices should have an absorption maximum matching the laser frequency, high basicity (p*K*_a_ > 10), and no acidic protons. In contrast, high acidity with minimal protonation in the gas phase seems to be an important characteristic of matrices designed for positive ion-mode analysis. Such simple matrix attributes can be calculated by using DFT methods. Jaskolla and coworkers represented another type of rational design of matrices [124]. In the experiment using various synthesized CHCA derivatives, beneficial halogenated substitution, such as 4-chloro-α-cyanocinnamic acid, leading to the reduction of proton affinity, induced a dramatic qualitative and quantitative improvement in MALDI performance. This study also suggested a rational of ion formation via chemical ionization mechanism with proton transfer from a reactive protonated matrix species to the analytes. Recently, Wang *et al.* reported that MALDI-FT-ICR-MS of porcine adrenal glands using quercetin, one of hydroflavones, led to determination of the spatial distribution of 555 unique endogenous compounds identified as 544 lipid entities and 11 nonlipid metabolites [125]. Quercetin showed characteristics superior to those of commonly used MALDI matrices—DHB, CHCA, and 2-mercaptobenzothiazole—which include: μm-sized matrix crystals, uniform matrix coating, low volatility in the high vacuum (~10^−7^ mbar) source, good chemical stability, low yield of matrix-related ions, low matrix consumption, low power threshold for laser desorption/ionization, and improved safety of handling [126]. These various types of information will play an indispensable role in the strategic development of MALDI-MS-based studies, including the choice of matrix and its design that will be the most effective for MALDI-MSI of endogenous metabolites, drugs, and food factors.

## 6. Conclusion and Perspectives

MSI has the promising capability for imaging small molecules, but there are many technical problems concerning ionization and ion separation. In conventional MS, analyte molecules are extracted and separated from crude samples by LC and GC, but this sample cleanup procedure is limited in MALDI-MSI, thus causing severe ion suppression effects, which are predominantly caused by competing numerous molecular species or by the presence of the highly abundant salts [116,127]. It is important to optimize the sample preparation condition so that the analyte molecules present in the crude mixture can be efficiently ionized [128]. In addition, researches on discovery and development of novel matrices [23,54,123,124,126] and alternative ionization methods [129,130,131,132] are also required for improving ionization efficiency of the target molecule. Furthermore, ion suppression and matrix crystallization effects are dependent on tissue composition and structure, and thus compromise quantitative performance of MSI. To overcome this issue, we must perform calculation of ion suppression effects and normalization to an endogenous or exogenous signal [87,116,133]. In low-*m/z* region, multiple ions from endogenous metabolites as well as matrix-related adduct clusters and fragments often share the same nominal mass [14,134]. To distinguish the target compounds of interest from such extensive chemical background, several important studies have been published. Tandem MS scanning [87,92,135] and its combination with ion mobility separation allow us to provide more selective target information [136,137,138]. Another approach is to use high-mass resolution and accuracy MS such as FT-ICR-MS [14,125] and Orbitrap MS [132]. To eliminate matrix-derived ions for effectively reducing the overlap of mass peaks from multiple compounds, organic matrix-free ionization methods have been developed, such as the use of nanoparticles [131,136], matrix-enhanced surface-assisted desorption/ionization [139], desorption/ionization on silicon [140], and nanostructure-initiator mass spectrometry [141]. 

Herein, we have discussed both the advantages and difficulties of the recent MALDI-MSI of endogenous metabolites or dietary phytochemicals. Improvement of the methods and development of instruments are still in progress in an effort to overcoming diverse technical disadvantages, such as molecular coverage and metabolite identification as well as match the highly sensitive quantification offered by labeling methods [67,88,142]. Implementation of both qualitative and quantitative MSI data for endogenous and exogenous molecular species of different classes has widespread impact on biological, pharmaceutical, and food functionality research. In the future, a combination of an *in situ* small-molecule MSI technique with other analytical platforms such as multivariate statistical analysis and *in vivo* non-invasive imaging techniques (for instance, anatomic imaging including MRI and computed tomography, or functional imaging including functional MRI and PET) may become the compulsory technology for unraveling and understanding the molecular complexities of local tissues or for *in situ* pharmacometabolomics, biomarker discovery, early diagnosis, and cytodiagnosis.
